# Regulation of Germinal Center, B-Cell Memory, and Plasma Cell Formation by Histone Modifiers

**DOI:** 10.3389/fimmu.2014.00596

**Published:** 2014-11-19

**Authors:** Kim L. Good-Jacobson

**Affiliations:** ^1^Immunology Division, Walter and Eliza Hall Institute of Medical Research, Parkville, VIC, Australia; ^2^Department of Medical Biology, University of Melbourne, Parkville, VIC, Australia

**Keywords:** humoral memory, B-cells, germinal centers, epigenetics, EZH2, MOZ, histone modifiers

## Abstract

Understanding the regulation of antibody production and B-cell memory formation and function is core to finding new treatments for B-cell-derived cancers, antibody-mediated autoimmune disorders, and immunodeficiencies. Progression from a small number of antigen-specific B-cells to the production of a large number of antibody-secreting cells is tightly regulated. Although much progress has been made in revealing the transcriptional regulation of B-cell differentiation that occurs during humoral immune responses, there are still many questions that remain unanswered. Recent work on the expression and roles of histone modifiers in lymphocytes has begun to shed light on this additional level of regulation. This review will discuss the recent advancements in understanding how humoral immune responses, in particular germinal centers and memory cells, are modulated by histone modifiers.

## Introduction

Pathogen clearance and formation of immunity requires the activation of B-cells and subsequent differentiation into antibody-secreting cells and memory cells. Humoral memory consists of both memory B-cells and long-lived plasma cells, the latter of which resides mainly in the bone marrow. Together, humoral memory cells are able to clear subsequent infections by the same pathogen more efficiently than responding B-cells during the initial response (1). The mechanisms underlying how memory is formed, and what controls its reactivation, are still unclear. In recent times, transcriptional regulation during B-cell differentiation (2–5) has been the focus of efforts to understand the intrinsic controls that regulate immune cell fates. In contrast, epigenetic regulation during a humoral immune response is relatively unknown. This review will discuss the limited information that is currently known about epigenetic regulators and their importance in the generation and maintenance of immune memory, focusing on the role of histone modifiers within the germinal center (GC).

## Humoral Immune Responses and Germinal Centers

**Figure 1:**
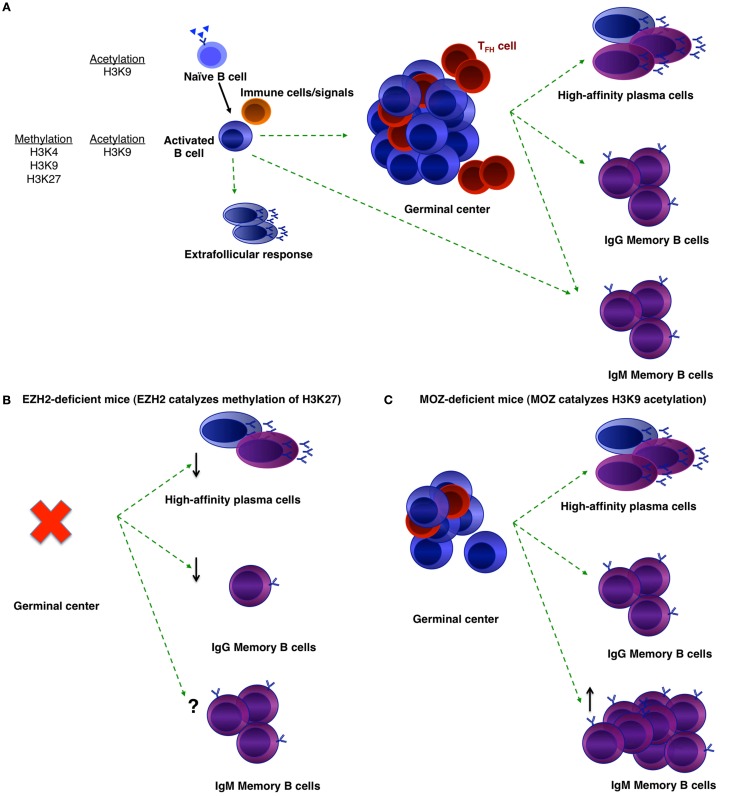
**Histone modifications that may regulate B-cell differentiation**. **(A)** B-cell differentiation during a T-dependent humoral response. Naïve B-cells that are specific to a foreign pathogen will become activated and receive help from accessory cells. They may become early memory B-cells or extrafollicular plasmablasts, or form a GC. GC B-cells then may either differentiate into long-lived plasma cells or memory B-cells. Within the memory population, there are IgM and IgG memory B-cells. Histone marks in naïve and activated B-cells are noted (15). **(B,C)** Phenotypes of EZH2-deficient **(B)** and MOZ-deficient **(C)** mice after immunization.

Humoral responses can be broadly categorized into either T-independent or T-dependent responses, with the production of high-affinity antibody and class-switched memory the main outcome of the latter (Figure 1). To this end, antigen-activated B-cells that receive T cell help and do not participate in the extrafollicular foci of low-affinity plasmablasts, or become early GC-independent memory (1, 6), can instead form GCs. GCs are divided into a light and dark zone. Within the dark zone, cells undergo multiple rounds of proliferation and adapt their antigen receptor to the immunizing antigen through the process of somatic hypermutation and class-switch recombination (7–11). B-cells then transition to the light zone, in which cells that have a high-affinity antigen receptor will be selected to continue to divide or to differentiate (12–14). In contrast, low-affinity cells and cells that have mutated their receptor to no longer be antigen-specific will die. High-affinity cells that are selected to survive may differentiate into plasma cells or memory cells.

Long-lived plasma cells are generally high-affinity, sessile cells that reside in the bone marrow, relying on extrinsic factors from niche cells for their survival (16). These plasma cells continually secrete high-affinity antibody, resulting in lowering of the amount of an invading pathogen upon re-encounter. Together with memory B-cells, they contribute to maintaining immunity.

## Regulation of Humoral Immune Responses

The activation, proliferation, and differentiation of antigen-activated B-cells during an immune response is orchestrated and regulated at both the cellular and molecular levels. During an immune response, B-cell behavior is regulated by both extrinsic and intrinsic mechanisms. B-cells respond to signals in the microenvironment, including cytokines, cell surface ligand/receptor pairings, and other soluble factors such as chemokines and cell survival molecules (17). For these signals to orchestrate cell behavior in a coordinated manner, cells integrate these signals, resulting in initiation or silencing of genes, which in turn directs cellular behavior.

Transcription factors are molecules that coordinate the expression of a number of genes, thus one transcription factor is often linked to the identity of a cell subset. Different B-cell subsets are associated with particular transcription factors. The transcriptional repressor B cell lymphoma 6 (BCL-6) is expressed in GC B-cells, regulating a program of genes required for GC function and as such is essential for the formation of GCs (18, 19). In contrast, the transcriptional repressor B lymphocyte-induced maturation protein 1 (Blimp-1) is expressed in plasma cells (20). BCL-6 and Blimp-1 were previously denoted as “master regulators” of B-cell differentiation, by reciprocally repressing each other (21). However, there are various lines of evidence demonstrating that, similar to the Th1/Th2 paradigm for T cells, the idea of master regulators was a useful concept but too simple to completely explain the genetic programs underlying B-cell differentiation (22). For example, in the case of memory B-cells, no master regulator transcription factor has been found, and both *Bcl-6* and *Prdm1*, the gene encoding Blimp-1, are downregulated (2). Furthermore, plasma cell differentiation can be induced in the absence of Blimp-1 (23).

Memory B-cells are the centerpiece of the secondary response, in which foreign pathogens are cleared more quickly than a primary response (1). As such, resting memory B-cells have decreased expression of cell cycle inhibitors, correlated with their ability to enter division earlier than naïve B-cells (4). Transcriptionally, naïve and memory B-cells are actually quite similar (3, 4), despite the enhanced survival and proliferative capabilities. Therefore, it is likely there is an additional level of regulation that endows memory B-cells with the ability to respond more efficiently to pathogen infection than naïve B-cells.

## Epigenetic Regulation

Genetic regulation also occurs via modification of histones. This is termed epigenetic regulation, i.e., stable inherited modifications of genetic material without altering the DNA sequence. The N-terminal tail of histones can be modified either to promote or inhibit transcription, via creating either an open chromatin structure (euchromatin) or a tightly packed structure (heterochromatin) (24). This is performed by histone modifiers, a group of enzymes such as methyltransferases, acetyltransferases, and histone deacetyltransferases (HDACs). Through these modifiers, histone structure and thus the ability of transcription to proceed is regulated (25, 26). DNA methylation is another form of epigenetic regulation, and recently it was demonstrated that inhibiting DNA methyltransferase 1 (DNMT1) can abrogate GC responses (27) (Table 1). However, due to space limitations, DNA methylation will not be discussed further here.

**Table 1:** **Humoral responses in the absence of EZH2, MOZ, p300 (acetyltransferase activity), or DNMT1 [from Ref. (27–31)]**.

Deletion	Type (target)	GC response	Memory	Plasma cells/Antibody
EZH2	Methyltransferase (H3K27)	– Absent – Reduction in proliferative cells – Higher frequency of cells in G0/G1 – Increase in apoptosis	Decreased IgG1^+^ memory and affinity	– Decreased IgG1, IgG2b – No change in IgG2a or IgG3 – Decreased plasma cells in vivo – Increased plasmablasts in vitro
MOZ	Acetyltransferase (H3K9)	– Decreased – Dark zone GC B-cells reduced Higher frequency of cells in G0/G1 Decreased BCL-6	– Numbers of IgG1^+^ memory normal but decreased affinity – Increased IgM^+^ memory	– No change in numbers, but decrease in affinity of plasma cells
p300^AT^	Acetyltransferase	No change	– Memory response impaired	– No change in IgG1 – IgG2b decreased – SLE-like disease
DNMT1	Methyltransferase (DNA)	– Decreased – Reduction in proliferative cells	Not assessed	Not assessed

In recent years, epigenetic regulation of B-cell development – especially VDJ recombination (32) – has been revealed. However, much less is known about whether epigenetic modifiers can regulate B-cell differentiation during a humoral response. This mini-review will focus specifically on the current understanding of differential histone modifications during the formation of GC-dependent memory.

## Histone Modification Patterns in Different B-Cell Subsets

Germinal center B-cells and plasma cells have their own unique transcriptional program compared to naïve and memory B-cells (2, 18–20). A large number of gene expression changes occur during differentiation of a naïve B-cell to GC to memory or plasma cell. In addition, an antigen-activated B-cell has the ability to choose any one of those three fates during a response. Therefore, it is likely that regulation of heterochromatin or euchromatin states plays a large role in this adaptability. It could be hypothesized then that the pattern of histone marks is unique to different mature B-cell subsets. Indeed, assessment of H3K4me1, H3K4me3, H3Ac, H3K36me3, H3K27me3, and PolII demonstrated that human naïve and GC B-cells had different patterns of open chromatin (33). Thus, it appears that there is a change in the epigenetic landscape either upon B-cell activation or during the first couple of days during an immune response.

## Changes to Histone Modifications upon Activation of B-Cells

The immediate epigenetic events that may occur upon activation of an antigen-specific B-cell are unknown. However, preliminary data have shown differences in histone marks between quiescent and activated B-cells (Figure 1). Methylation of various histone lysines was found to be decreased in resting cells compared to *in vitro* activated cells (15). For example, H3K4, H3K9, and H3K27 methylation increased after *in vitro* activation, whereas, H3K9 acetylation is present in both quiescent and activated cells (15). In contrast to H3K9 and H3K27 methylation, H3K4 methylation is a permissive mark. Although the authors suggest that histone lysine hypomethylation was a mechanism that endowed B-cells with reprograming potential (15), this has yet to be shown functionally.

Although it is clear that different B-cell subsets have different patterns of histone modifications, there is limited evidence on the role particular histone modifiers play during the early phase of humoral responses. For example, B-cells from a mouse engineered to have reduced acetyltransferase activity in p300 were still able to respond to T cell-derived stimuli such as anti-CD40, IL-4, and the T-independent stimuli LPS or CpG agonist (28). In contrast, there was a 50% reduction in the ability of these cells to respond to BCR stimulation (28). Because B-cell development is altered in these mice, it is not clear whether this defect is the result of a defect that occurred during B-cell development, as opposed to a direct role upon BcR engagement in the periphery.

An area of great interest currently is whether “bivalency,” i.e., the presence of both activating and repressive marks at the same loci, is important for lymphocyte plasticity in identity and function (22). Preliminary studies suggest that bivalency is an important regulator of gene expression during differentiation of naïve to GC B-cells. Enhancer of zeste homolog 2 (EZH2) is a histone methyltransferase and a polycomb group member that catalyzes methylation of H3K27 (34). A number of EZH2 target genes in centroblasts that were marked by H3K27me3 were also H3K27me3 marked in naïve B-cells, although likely not by EZH2 as its expression is very low in naïve B-cells (35). A study of bivalent genes in naïve and GC B-cells (with respect to the activating mark H3K4me3 and silencing mark H3K27me3) found that differentiation into GC B-cells resulted in ~1000 new bivalent domains (29). However, the vast majority of these promoters that had dual marks came from the acquisition of H3K27me3 (likely due to upregulation of EZH2) – i.e., already marked H3K4me3 promoters in naïve B-cells (29). As the transcriptional program in GCs is known to involve the large-scale repression of many genes, bivalency may allow GC B-cells to establish the transcriptional program required for the multiple rounds of proliferation and somatic hypermutation that occurs, while retaining the ability to differentiate into centrocytes and eventually plasma cells and memory B-cells. However, the likely complex roles of bivalent domains during B-cell differentiation are yet to be unraveled.

## Regulation of GCs by EZH2 and MOZ

Polycomb group proteins are differentially expressed in the GC in human tonsils. BMI-1 and RING1 downregulation, and ENX and EED upregulation, occur upon differentiation into centroblasts (36). This was then reversed in centrocytes. EZH2 was also found to be upregulated in centroblasts (30, 35, 37). It has also been shown that while *Ezh2* is expressed in plasmablasts, BMI-1 is expressed in plasma cells (38), correlating EZH2 expression with cycling cells in both the GC and in the plasmablast populations. The expression of *Ezh2* is decreased, however, in PC and memory B-cell populations compared to GC B-cells (30).

To investigate the role of epigenetic regulation in B-cell differentiation during humoral responses, a number of groups have conditionally deleted histone modifiers (Table 1). Two such enzymes are EZH2 and the histone acetyltransferase monocytic leukemia zinc finger protein (MOZ) (Figure 1). EZH2 plays an important role during B-cell development by modulating *Igh* rearrangement (39), and has now been revealed to be essential for GCs (29, 30). The deletion of EZH2, by use of either Cγ1-Cre or CR2-Cre, dramatically reduced GC frequency, with the remaining GC cells EZH2^+^ (29, 30). Both research groups demonstrated the regulation of cell cycle genes by EZH2 (29, 30, 35), although GC B-cells were also found to undergo increased apoptosis in the absence of EZH2 (30).

Although MOZ is a histone acetyltransferase, there were similarities between the phenotypes of MOZ-deficient and EZH2-deficient mice. Deletion of MOZ using *Mb-1*-Cre (in all B-cells) or *Aicda*-cre (specifically in activated B-cells) mice also resulted in a decrease in GC B-cells (31), although not to the extent of EZH2-deficiency (29, 30). This was found to be due to defective proliferation, correlating to a decrease specifically in dark zone B-cells (31). Thus, expression and/or function of EZH2 and MOZ can be localized to the dark zone of the GC. Somatic hypermutation and class-switch recombination is also known to be regulated epigenetically, however, this has been reviewed recently (32) and thus will not be discussed here. Given that a number of other histone modifiers are located either in the dark or light zone (36, 37), future investigations could assess whether these other modifiers regulate particular functions within the light zone.

B cell lymphoma 6 (BCL-6) is absolutely required for GC formation (18, 19). BCL-6 shares some common targets with EZH2 in GC B-cells. EZH2 binds approximately 1800 promoters in GC B-cells (35), and a portion of these were specific to GC B-cells. Within this GC-specific geneset, it appeared that EZH2 targets were involved in cellular proliferation and repression of differentiation (29, 30, 35). Interestingly, EZH2 targets that were not H3K27me3-marked in naïve B-cells were also bound by BCL-6 (35). Approximately half of the genes that were bound by both the polycomb repressor complex 2 and BCL-6 in wild-type GC B-cells were upregulated in EZH2 mutants (30). In contrast, EZH2-deficiency mostly did not affect the expression of BCL-6 targets that lack the H3K27me3 mark (30), and EZH2 does not modulate BCL-6 expression itself (30). In contrast, MOZ-deficient GC B-cells had decreased levels of BCL-6 (31), which may be associated with the gene expression program modulated by MOZ (31).

## Regulation of Plasma Cells by Histone Modifiers

Conditional deletion of histone modifiers in B-cells has demonstrated that differentiation of GC cells into plasma cells is epigenetically regulated. In the case of MOZ, deficiency altered the affinity but not numbers of plasma cells, likely due to the reduction of dark zone B-cells (31). Similarly, the GC defect in EZH2-deficient mice resulted in a significant reduction in both numbers and affinity of plasma cells (30). However, when these authors stimulated EZH2-deficient cells *in vitro*, differentiation into plasmablasts was increased in the absence of EZH2. This was correlated to functional repression of the plasma cell genes *Prdm1* and *Irf4* by EZH2 (30), and the reduction of H3K27me3 marking at *Irf4* and *Prdm1* loci upon differentiation (30). In addition to *Irf4* and *Prdm1*, EZH2 appears to regulate the genetic programs associated with differentiation of GC B-cells to plasma cells or memory B-cells (29, 30, 35). Thus, continued EZH2 expression is likely required to maintain the GC phenotype and prevent premature differentiation (35). It is known that EZH2 mutations are associated with malignant transformations (29, 30, 35, 40), but it is also possible that dysregulation of EZH2 may also play a role in antibody-mediated autoimmune disorders.

Lastly, it is likely that HDACs can also regulate plasma cell differentiation, although previous studies have had contrasting results on whether inhibiting HDACs inhibit or promote differentiation (41, 42). This will be important to determine as HDAC inhibitors are being used to treat lymphocyte malignancies (43–45). Dysregulation of gene expression during B-cell responses can lead to autoimmune diseases, and there is some evidence this could occur as a result of improper histone modifications. Mice lacking acetyltransferase activity in p300 specifically in B-cells develop a systemic lupus erythematosus-like disease (28). Thus, there is future potential to use epigenetic modifiers as treatment for antibody disorders.

## Regulation of B-Cell Memory by EZH2 and MOZ

Immune memory is defined as the rapid and robust response that occurs upon secondary infections, clearing invading pathogens more quickly than the primary response. The memory B-cell population is phenotypically and functionally heterogeneous (1, 46, 47). Recently, a number of research groups have postulated that the heterogeneity evident within the memory population allows the pool to undergo specialized functions, i.e., differentiation into plasmablasts whilst being able to self-renew. IgM^+^ memory B-cells persist longer than IgG^+^ memory B-cells, and are able to initiate a response to secondary infections when IgG^+^ memory B-cells are present in low numbers (48, 49). In contrast, switched memory B-cells has been linked to the rapid production of antibody during secondary responses (48, 49). A number of genes expressed in IgM^+^ B-cells are silenced when those cells are engineered to signal through the cytoplasmic tail of IgG1 (50, 51). Therefore, regulation of gene transcription programs may be linked with the plasticity of the memory pool, allowing persistence in the presence of rapid activation and differentiation during re-infection.

In the absence of EZH2, GC-derived IgG1^+^ memory B-cells and antibody produced in a secondary response were significantly reduced (30). It is likely that the reduction in memory formation and function is a result of the absence of functional GCs (29, 30). High affinity IgG1^+^ memory B-cells were also reduced in the absence of MOZ (31). The latter study also investigated the IgM^+^ memory B-cell subset, which has been linked to longevity of the memory population (48, 49). In the absence of MOZ, the make-up of the memory B-cell population was altered such that IgM^+^ memory B-cell numbers were increased. It is likely that as a result, secondary GC formation was increased in these mice, whereas, secondary plasmablast formation was decreased (31). Thus, MOZ regulated the composition and functional outcome of the memory compartment. More work is now needed to investigate in detail the role of epigenetic regulation in memory B-cell formation and function.

## Concluding Remarks

Histone modifications are an important component of gene expression regulation. Specifically, in both T and B-cells, during development and during differentiation in the periphery, patterns of histone modifications are unique to different lymphocyte subsets. These modifications likely allow adaptability of cells – i.e., for the ability of an antigen-activated B-cell to undergo differentiation into either a memory B-cell, GC or plasmablast.

The enzymes that catalyze modifications of histones, such as EZH2 and MOZ, have recently been shown to play important roles in formation, maintenance and modulation of B-cell populations. Thus, these new studies demonstrate that programing of B-cell subsets by epigenetic changes influence differentiation decisions during immune responses. However, it is only the beginning for these types of studies. A better understanding of epigenetic regulation of humoral responses will be attained as the targets for each modifier in B-cell subsets, factors involved in facilitating modifications, and interactions between known regulatory complexes are revealed. It will be important to use an integrated approach to identify histone modifications important for B cell generation and function, and the transcriptional networks they regulate. Thus, in addition to ChIP-sequencing and gene-targeted mice, it will be essential to use new methods that can systematically initiate histone marks during B cell responses to unravel the role of particular modifications during memory formation and secondary responses.

Revealing the roles of other histone modifiers has the potential to reveal the molecular mechanisms underlying the production of a memory population that is able to persist in the absence of antigen whilst being poised to respond to subsequent infections. This not only has implications for vaccines and immunodeficiencies that are unable to produce memory cells, it will also result in a wider understanding of how epigenetic regulation controls gene expression during programs of cell differentiation. Understanding these fundamental cellular processes are applicable not only to B-cell and hematopoietic development, but also more generally for developmental processes. It is noteworthy that it is precisely these transcriptional networks that are predictive in disease, particularly autoimmune diseases and cancers.

## Conflict of Interest Statement

The author declares that the research was conducted in the absence of any commercial or financial relationships that could be construed as a potential conflict of interest.
